# Prognostic value and immune infiltration of the gasdermin family in lung adenocarcinoma

**DOI:** 10.3389/fonc.2022.1043862

**Published:** 2022-11-25

**Authors:** Lu-Shan Peng, Sai-Li Duan, Run-Qi Li, Dan Wang, Ying-Ying Han, Tao Huang, Yu-Pei Yu, Chun-Lin Ou, Jun-Pu Wang

**Affiliations:** ^1^ Department of Pathology, Xiangya Hospital, Central South University, Changsha, Hunan, China; ^2^ Department of General Surgery, Xiangya Hospital, Central South University, Changsha, Hunan, China; ^3^ Department of Pathology, School of Basic Medicine, Central South University, Changsha, Hunan, China; ^4^ National Clinical Research Center for Geriatric Disorders, Xiangya Hospital, Central South University, Changsha, China; ^5^ Key Laboratory of Hunan Province in Neurodegenerative Disorders, Xiangya Hospital, Central South University, Changsha, Hunan, China

**Keywords:** GSDM family, lung adenocarcinoma, methylation, immune infiltration, biomarker, prognosis

## Abstract

**Background:**

The GSDM family includes six members, GSDMA, GSDMB, GSDMC, GSDMD, GSDME (DFNA5), and PJVK (Pejvakin, DFNB59), which can induce pyroptosis, thereby regulating the tumorigenesis of various cancers. However, the clinical characteristics and role of the GSDM family in LUAD are not well understood.

**Methods:**

In this study, several important bioinformatics databases were used to integrate the analysis of the expression, prognostic value, and immune infiltration of GSDMs in LUAD. These databases include UALCAN, DiseaseMeth, GEPIA, THPA, cBioPortal, TIMER, WebGestalt, STRING database, and Cytoscape.

**Results:**

The findings from the UALCAN database revealed that the expression of all six GSDMs based on the tumor stage in LUAD was increased (particularly GSDMD). Our IHC results verified it. Additionally, the DiseaseMeth database showed that the methylation levels of GSDMA, GSDMB, GSDMC, and GSDMD were decreased. The expression of six GSDMs was related to shorter overall survival in patients with LUAD, according to the GEPIA database. The cBioPortal database was further used to explore the alteration rate and correlated genes in LUAD. Subsequently, these genes were subjected to functional enrichment and protein-protein interaction network analyses. We identified that the GSDM family regulate several signaling pathways, including immune-associated signaling pathways. According to tumor-infiltrating immune cell analysis from the TIMER database, GSDM family members are associated with the infiltration of important immune cells and their signature markers.

**Conclusions:**

GSDM family may be prognostic markers and novel strategies for the treatment of LUAD.

## Introduction

Lung cancer is the leading cause of cancer-related deaths (18.0%) among the top ten cancer types causing over 1.7 million deaths worldwide in 2020 (1). Lung adenocarcinoma (LUAD) recently surpassed lung squamous cell carcinoma as the most common lung cancer subtype (2), accounting for 30%–35% of the total number of lung cancer cases. Consequently, novel biomarker exploration may be necessary for diagnosing and treating LUAD (3).

In humans, there are six gasdermins (GSDMs), including GSDMA, GSDMB, GSDMC, GSDMD, GSDME (DFNA5), and PJVK (Pejvakin, DFNB59). In addition to PJVK, the other five GSDMs promote cell death mainly through pyroptosis, a lytic and pro-inflammatory form of cell death (4). Pyroptosis is also known as “gasdermin-mediated programmed cell death” (5). In the past, cell death was mainly divided into apoptosis and necrosis. In recent years, scientists have proposed that cell death also involves other forms of programmed necroptosis, including pyroptosis (6).Pyroptosis can be accompanied by immune responses and the release of pro-inflammatory factors, which play a vital role in various genetic diseases, autoinflammatory diseases, and tumors (7, 8). A recent study found that GSDMs are a family of histones that play a decisive role in pyroptosis (9). They can induce inflammatory responses, assist the transition from apoptosis to pyroptosis (10, 11), and play an important role in the occurrence and development of various tumors (12, 13). For example, Zhou et al. found that gasdermin-mediated pyroptosis may enhance antitumor immunity through pyroptosis-induced inflammation (14, 15). Similar function was suggested in GSDMB and GSDME (16) (17) (18). However, the exact mechanism of the role of GSDM family tumors is not clear. There are also reports on GSDMs family members about their cancer-promoting effect. GSDMD could be used as a prognostic marker in different cancers, including adrenocortical carcinoma, kidney renal clear cell carcinoma, brain lower grade glioma, hepatocellular carcinoma, skin cutaneous melanoma, and rectal adenocarcinoma (19).High GSDMC expression was significantly associated with the tumorigenesis of clear cell renal cell carcinoma (20). In LUAD, a study found that high GSDMD expression may promote the occurrence and development of the tumors. This indicates a poor prognosis of LUAD and may serve as an independent prognostic biomarker (21). Upregulated GSDMC expression is an independent indicator of poor first progression (FP) and poor overall survival (OS) in patients with LUAD, which is regarded as a promising predictor of poor prognosis (22). Therefore, the GSDM family might play a role in promoting tumorigenesis in LUAD. In LUAD, there are only 2 researches, both of them believe that GSDM could promote the development of LUAD. A study found that high GSDMD expression may promote the occurrence and development of the tumors. This indicates a poor prognosis of LUAD and may serve as an independent prognostic biomarker (21). Upregulated GSDMC expression is an independent indicator of poor first progression (FP) and poor overall survival (OS) in patients with LUAD, which is regarded as a promising predictor of poor prognosis (22). We think different role of GSDM family members toward tumor is related to the heterogeneity of tumors. Subsequently, we speculated that the GSDM family might play a role in promoting tumorigenesis in LUAD. However, detailed, and systematic studies on the role of the GSDM family in LUAD is lacking.

In the present study, the function of GSDMs in LUAD was analyzed using several bioinformatics databases, such as gene expression profiling interactive analysis (GEPIA), UALCAN, and cBioPortal to explore the role of GSDMs in LUAD, which might help identify the role of GSDMs in LUAD and provide new strategies for LUAD treatment.

## Methods

### GEPIA

We use Gene Expression Profiling Interactive Analysis (GEPIA), a cancer big data analysis website based on TCGA and GTEx data, to assess the expression of the GSDMs family in LUAD tissues and in normal tissues. The analysis contents include tumor/normal differential expression profiling, expression distribution, pathological staging, survival analysis, similar genes, gene expression correlation and dimensionality reduction analysis, etc. (23). Also, the roles of GSDMs family in pathological stage and prognosis are evaluated by using the GEPIA.

### The human protein atlas

The Human Protein Atlas (HPA) is a useful database for studying protein localization and expression in human tissues and cells, which contains more than 10 million images (24). By using this database, we obtain the immunohistochemistry images of all six GSDMs family members in LUAD and in normal tissues.

### UALCAN

UALCAN is an interactive web-portal which perform in-depth analyses of TCGA gene expression data. TCGA level 3 RNA-seq and 31 cancer types clinical data are used in this database (25). We use this database to assess the mRNA expression of six GSDMs family members according to the LUAD stage, and the mRNA expression of six GSDMs family members in different LUAD histology subtypes.

### cBioPortal

The cBioPortal is a prevalent open-source translational research platform, providing genomics from more than 200 cancers (26, 27). We use Cbioportal to explore the coexpression profiles and genetic alterations of the GSDMs family in LUAD.

### WebGestalt

WebGestalt is a tool which can interpretate the gene lists derived from large scale -omics studies. This database is often used for functional enrichment analysis (28). We use this database to analyze the functional enrichment of GSDMs co-expression genes.

### Timer

Timer is a comprehensive database with visualization functions of tumor infiltrating immune cells (29). In this study, Timer was used to get the scatterplots that show the correlation of GSDMs with 6 types of infiltrated immune cells including dendritic cells, macrophages, CD4+ T cells, CD8+ T cells, B cells, and neutrophils, and their signature markers.

### DiseaseMeth

DiseaseMeth is a database including DNA methylation data in 162 diseases and 4,9 949 High-throughput profiles samples (30). We use this database to explore the methylation levels of the GSDMs family members.

### STRING and Cytoscape

STRING is a database which can be used to explore all publicly available sources of protein-protein interaction information, and to complement these with computational prediction (31). Cytoscape is an open-source platform for network analysis and visualization. We use STRING and Cytoscape together to construct the PPIs network.

### Immune infiltration analysis by ssGSEA

The analysis of NK cell infiltration in LUAD was performed by single sample GSEA (ssGSEA) method from R package GSVA (version 3.6.3) (https://portal.gdc.cancer.gov/). P values were determined by the Spearman and Wilcoxon rank sum test. We think P values < 0.05 were statistically significant.

### Immunohistochemistry

10 LUAD specimens were achieved from the department of pathology, Xiangya Hospital, Central South University. All specimens were fixed in 10% neutral-buffered formalin, embedded in paraffin, and stained with hematoxylin and eosin for histological examination. During initial case diagnosis, immunohistochemical staining was performed by using formalin-fixed paraffin-embedded tissue sections and the Ventana Benchmark automated immunostainer (Ventana Medical System Inc, Roche, Tucson, AZ, USA). We used GSDMD (Proteintech, #20770–1-AP, 1:200) as primary antibodies, and incubated with it overnight at 4 °C. The sections were then incubated with the secondary antibodies and DAB regents for staining (Zhong Shan Golden Bridge Biotechnology, Beijing, China).

## Results

### Differential expression of GSDMs between tumorous and adjacent normal tissues

**Figure 1:**
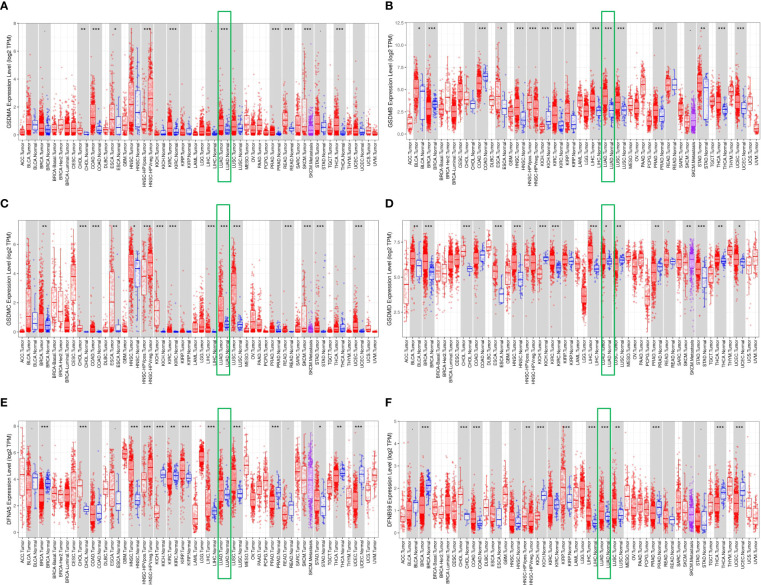
Differential expression of GSDMs between tumor and adjacent normal tissues. The expression of **(A)** GSDMA, **(B)** GSDMB, **(C)** GSDMC, **(D)** GSDMD, **(E)** GSDME, and **(F)** PJVK in different tumors and normal tissues based on the Timer database. * means p<0.05, ** means p<0.005, and *** means p<0.001.

First, the TIMER database was used to compare the expression levels of six GSDMs in cancerous and normal tissues. It was found that GSDMs were overexpressed in several tumors, including cholangiocarcinoma, bladder urothelial, kidney renal clear cell carcinoma, and LUAD (**Figure 1**). Among them, we found that in LUAD, all six GSDM family members are upregulated.

### Abnormally-increased expression of GSDM family members in LUAD

**Figure 2:**
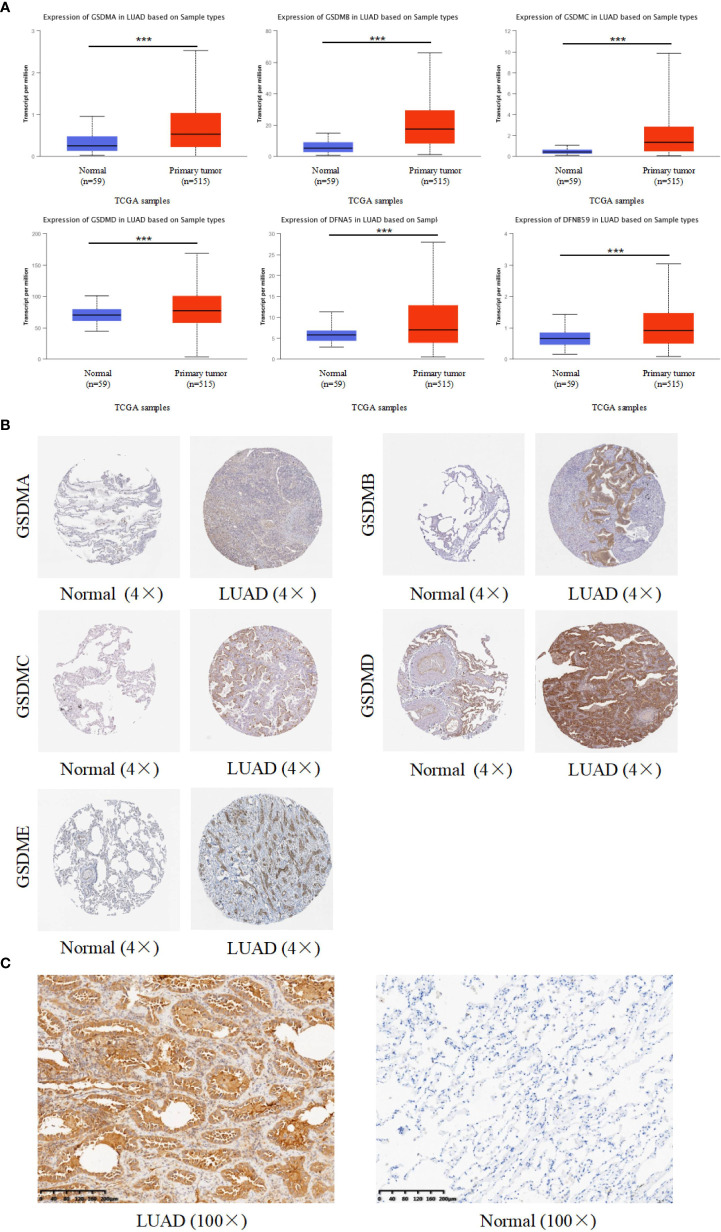
Expression of six GSDM family members in LUAD. **(A)** The UALCAN database was used to retrieve the GSDMs’ mRNA expression profiles. **(B)** The THPA database was used to collect IHC images of GSDM family members from LUAD tissues and normal lung tissues, representing the GSDM protein expression levels. **(C)** The IHC result of GSDMD expression in LAUD and adjacent normal lung tissue. *** means p<0.001.

For targeted research on LUAD, the UALCAN database was used to evaluate mRNA expression levels of the GSDM family in normal and LUAD tissues. The mRNA expression levels of all six GSDM family members were higher in LUAD tissues than in normal tissues. Among the six members, the expression level of GSDMD was the highest, whereas that of GSDMC was the lowest (**Figure 2A**). The Human Protein Atlas database (THPA) was used to analyze the protein expression of GSDMs in LUAD and normal tissues through the immunohistochemistry (IHC) staining. The results showed that the expression levels of GSDMA, GSDMB, GSDMC, GSDMD, and GSDME were increased in LUAD tissues (**Figure 2B**), especially GSDMD, which was significantly increased. The IHC data related to PJVK were not retrieved from the THPA database. Collectively, the expression of GSDMs was significantly high (P < 0.005) in LUAD tissues. To verify the expression of GSDMs in LUAD. We made IHC of GSDMD in 10 specimens (due to the result of the expression of GSDMD was the highest. The result supported the result retrieved from database (**Figure 2C** **)**.

### Clinical-pathological characterizations in patients with LUAD

**Figure 3:**
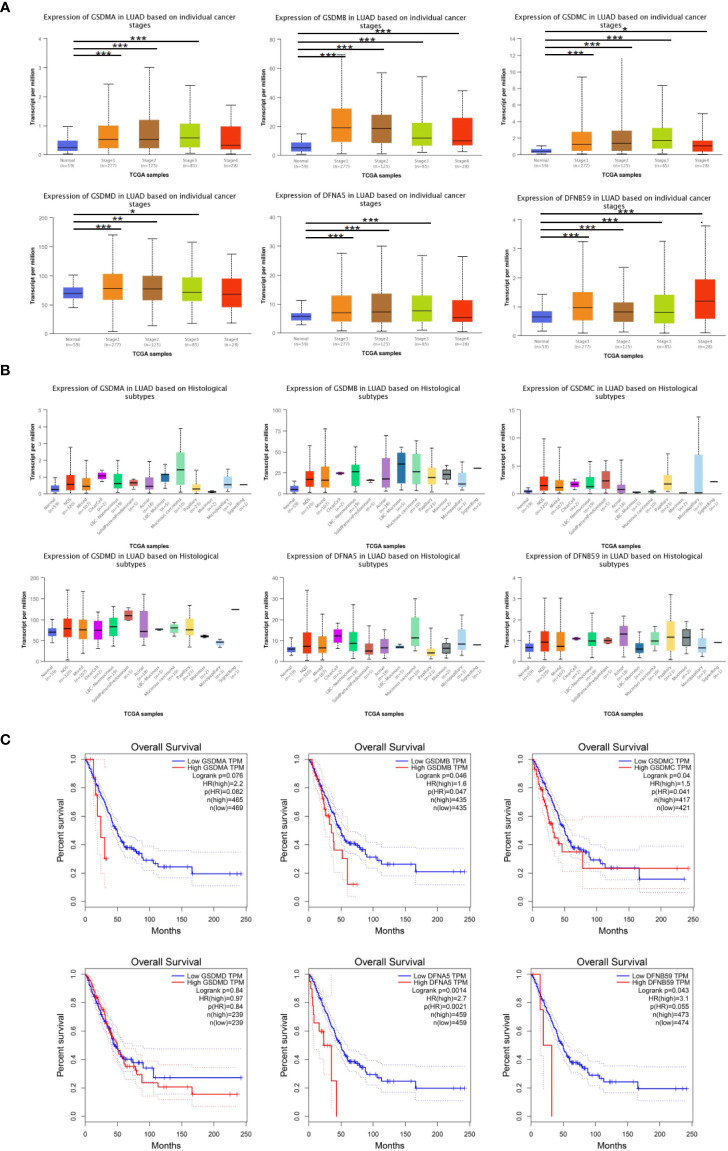
GSDMs family member-associated clinical pathology characteristics of patients with LUAD. **(A)** The associations between GSDM transcript levels and individual cancer stages of LUAD. **(B)** The expression of GSDM family members in different LUAD histological subtypes. **(C)** The GEPIA database was used to analyze the relationships between GSDM transcript levels and OS of LUAD patients. * means p<0.05, ** means p<0.005, and *** means p<0.001.

Furthermore, the influence of GSDMs on clinical-pathological characterizations in patients with LUAD was elucidated. The tumor stage describes the severity of malignant tumors according to the primary tumor and its degree of dissemination inside the body, which helps understand the prognosis and outcome of the disease. Based on the analysis of UALCAN, the expression of all GSDM family members was increased in tumor-stage 1–3 subgroups, and the expression of GSDMA, GSDMB, GSDMC, and PJVK was higher in all four tumor-stage subgroups than in normal subgroups (**Figure 3A**). This indicate that the abnormal expression of GSDMs may be involved in the progression of LUAD through certain mechanisms. Furthermore, the expression of six GSDMs based on LUAD histological subtypes was analyzed (**Figure 3B**). The results showed that the expression of GSDMs was elevated in most of the LUAD histological subtypes. This further suggests that the abnormal expression of GSDMs has an important influence on the clinicopathological features of different types of LUAD.

In addition, the GEPIA database was used to explore the prognostic role of the GSDM family in patients with LUAD. The overall survival (OS) curves and interactive correlations are presented in **Figure 3C**. This analysis suggested that, in patients with LUAD, high expression of GSDMs was significantly related to shorter OS, except for GSDMC (P < 0.05). These results suggest that GSDMs could provide prognostic information in patients with LUAD.

### Genetic alteration and methylation level analyses of GSDM family in patients with LUAD

**Figure 4:**
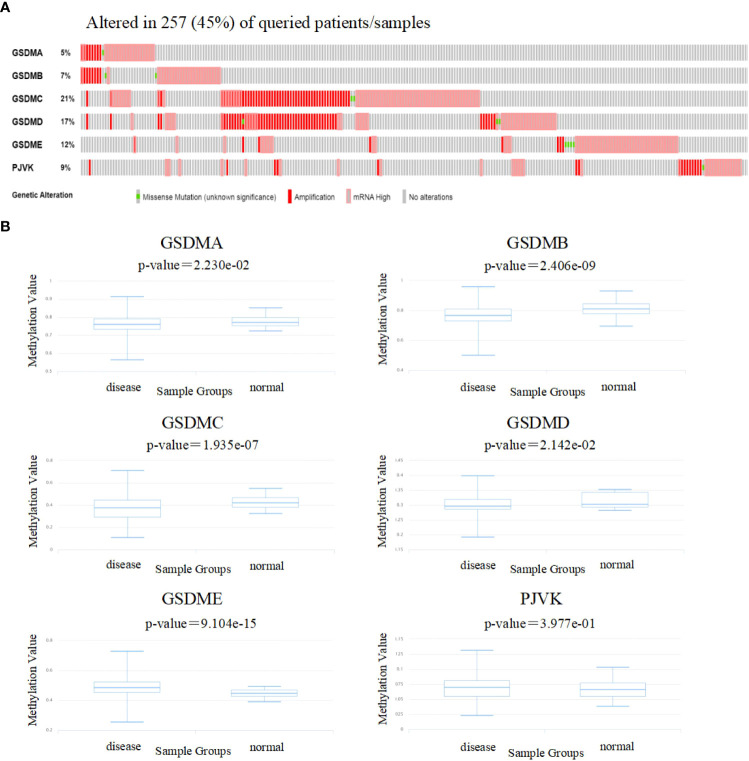
Genetic alterations and methylation levels of GSDM family members in LUAD. **(A)** The cBioPortal database was used to achieve the genetic alteration profiles of GSDMs. **(B)** The DiseaseMeth database was used to evaluate the DNA methylation levels of GSDM family members in LUAD.

To gain insight into the influence of GSDMs on LUAD. genetic alterations in GSDMs were analyzed (**Figure 4A**). Among the 566 enrolled patients with LUAD, 257 (45%) had altered GSDM gene expression. The highest and lowest genetic alteration rates were 21% (GSDMC) and 6% (GSDMA), respectively. Amplification and high mRNA expression are the two main types of alterations in the GSDM family members.

Next, the DiseaseMeth database was used to evaluate the methylation levels of the GSDMs (**Figure 4B**). Several studies have shown that abnormal DNA methylation is closely associated with carcinogenesis and tumorigenesis (32, 33). Thus, oncogenes may be activated by reduced methylation levels. In this study, we found that the methylation levels of GSDMA, GSDMB, GSDMC, and GSDMD were lower in LUAD tissues compared to that in normal tissues, which may be associated with their abnormal expression in LUAD.

Based on this, the genes correlated with GSDMs were further explored using the cBioPortal database. The results showed that there were 19316 genes correlated with GSDMs.

### Functional enrichment analysis of the GSDMs-associated-coexpressed genes

**Figure 5:**
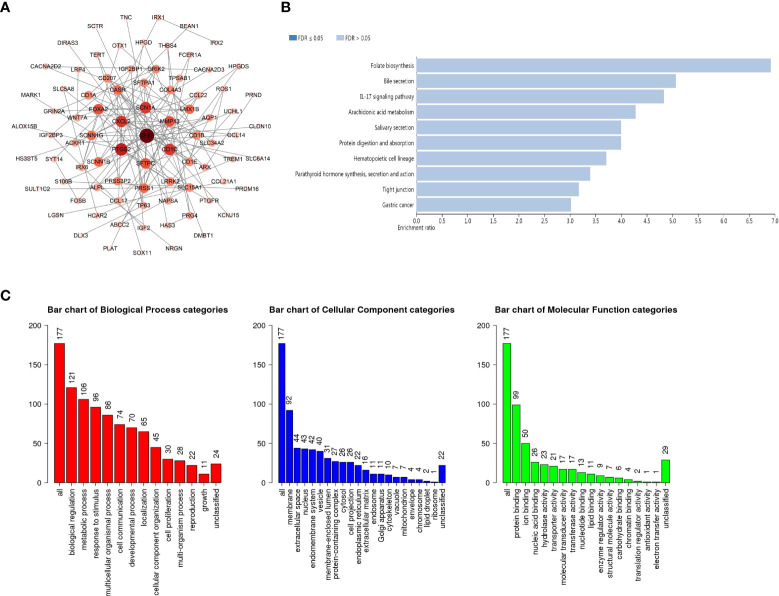
PPI network and the biological pathways of GSDM family members. **(A)** STRING and Cytoscape were used to build the PPI network. The WebGestalt database was used for the GSDM-related co-expressed molecules to analyze the GO functional enrichment analysis **(B)** and the KEGG pathway analysis **(C)**.

To further explore the role of GSDMs in LUAD, functional analysis was conducted. A total of 19316 correlated genes were downloaded, 177 of which were screened according to |log ratio| >0.8 and p<0.05, using the cBioPortal database (**Supplementary Table 1**). Based on this, the protein-protein interaction (PPI) network was constructed using the STRING database and Cytoscape (**Figure 5A**). Furthermore, the WebGestalt database was used to conduct Kyoto Encyclopedia of Genes and Genomes (KEGG) enrichment analysis. It was found that there were ten potential signaling pathways, and folate biosynthesis, bile secretion, and IL-17 signaling pathways were ranked in the top three, indicating that these pathways may be strongly associated with the progression of LUAD (**Figure 5B**). Next, Gene Ontology (GO) annotation from the same database (**Figure 5C**) suggested that GSDMs were mainly enriched in the membrane. Biological regulation was the most enriched biological process category. In addition, metabolic processes, responses to stimuli, multicellular organismal processes, localization, cell communication, cellular component organization, and developmental processes were also highly enriched.

### Immune cell infiltration of the GSDMs family

**Figure 6:**
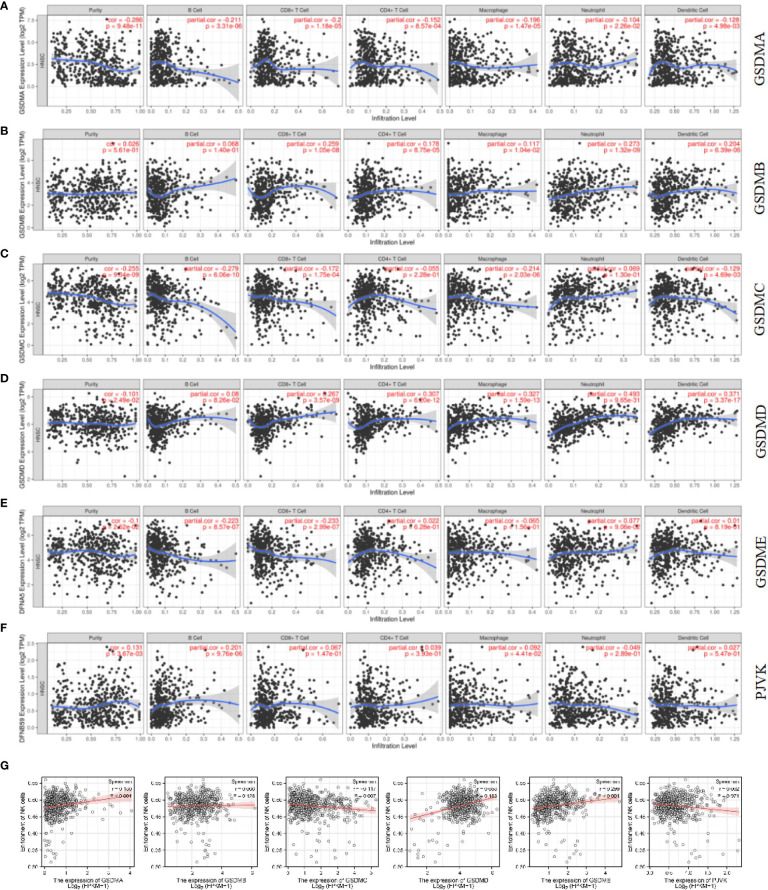
Correlation between immune cell infiltration and GSDM mRNA expression levels. The TIMER database was used to evaluate the association of GSDMA **(A)**, GSDMB **(B)**, GSDMC **(C)**, GSDMD **(D)**, GSDME **(E)**, and PJVK **(F)** expression and immune cell infiltration. The ssGSEA method from R package GSVA was used to evaluate the association of six GSDM family members and NK cell **(G)**.

In recent years, immune cells have been considered a crucial part of the tumor microenvironment, which is the interval environment for tumor initiation and survival (34, 35). The functional enrichment analysis suggested that some pathways and biological processes of GSDMs are related to immunity. Therefore, the Timer database was used to explore the correlation between tumor-infiltrating immune cells and the GSDM family. This study revealed that the expression of GSDMA and GSDME was positively correlated with the infiltration of all six immune cells, including B cells, CD8^+^ T cells, CD4^+^ T cells, macrophages, neutrophils, and dendritic cells (**Figures 6A, E**). GSDMB was positively correlated with the infiltration of B cells (Cor = 0.232, p < 0.05), CD4^+^ T cells (Cor = 0.318, p < 0.05), and dendritic cells (Cor = 0.108, p < 0.05) (**Figure 6B**). However, GSDMC and the infiltration of different immune cells were not closely correlated (**Figure 6C**). GSDMD was found to be negatively correlated with the infiltration of CD8^+^ T cells (Cor = -0.109, p < 0.05) but positively correlated with the infiltration of B cells (Cor = 0.223, p < 0.05) and CD4^+^ T cells (Cor = 0.276, p < 0.05) (**Figure 6D**). **Figure 6F** shows that PJVK was positively correlated with CD4^+^ T cells (Cor = 0.122, p < 0.05), whereas it was negatively correlated with other immune cells, except B cells. Natural killer cell (NK) is an important immune cell and closely related with anti-tumor response. Unlike T and B cells, NK cells are a class of lymphocytes that can non-specifically kill tumor cells without pre-sensitization. We analyzed the infiltration of NK cells in LUAD by using ssGSEA (**Figure 6G** **)**. We found that GSDMC was negatively correlated with NK cells (p=0.007). While GSDMA (p < 0.001) and GSDME (p < 0.001) were found positively correlated with NK cells. These results indicated a potential relationship between the role of GSDMs in LUAD and the infiltration of different immune cells.

Additionally, the relationship between GSDMs and signature markers of various immune infiltrating cells in LUAD was explored using the TIMER database (**Table 1**). The results showed that, among the six members, the expression of GSDMA, GSDMB, GSDMD, and GSDME was significantly correlated with most signature marker genes of various immune cells in LUAD (GSDME expression was negatively correlated).

**Table 1:** Association between the expression of GSDM family members and the markers of immune cells.

Description	Markers	GSDMA		GSDMB		GSDMC		GSDMD		GSDME		PJVK	
		Cor.	*P*	Cor.	*P*	Cor.	*P*	Cor.	*P*	Cor.	*P*	Cor.	*P*
CD8+ T cell	CD8A	0.011	7.99e-01	0.045	3.07e-01	0.027	5.46e-01	0.158	3.13e-04	0.196	7.4e-06	-0.091	3.83e-02
	CD8B	-0.053	2.23e-01	0.073	9.78e-02	-0.006	8.92e-01	0.212	1.25e-06	0.172	9.16e-05	-0.091	3.84e-02
	GZMA	-0.049	2.59e-01	0.059	1.81e-01	0.042	3.4e-01	0.155	4.4e-04	0.184	2.68e-05	-0.115	9.05e-03
B cell	CD19	-0.092	3.41e-02	0.147	8.12e-04	0.02	6.55e-01	0.19	1.42e-05	0.112	1.07e-02	0.102	2.06e-02
	CD79A	-0.122	4.92e-03	0.041	3.52e-01	-0.008	8.49e-01	0.105	1.71e-02	0.123	5.23e-03	-0.023	6.1e-01
	MS4A1	0.108	1.26e-02	0.171	9.97e-05	0.021	6.29e-01	0.159	3.01e-04	0.115	8.77e-03	0.135	2.16e-03
CD4+ T cell	CD2	0.004	9.24e-01	0.168	1.26e-04	0.007	8.66e-01	0.21	1.6e-06	0.263	1.46e-09	-0.029	5.12e-01
	CD3D	-0.083	5.41e-02	0.142	1.27e-03	0	9.92e-01	0.227	2e-07	0.226	2.08e-07	-0.069	1.17e-01
	CD3E	-0.03	4.85e-01	0.152	5.28e-04	0.003	9.55e-01	0.197	6.39e-06	0.252	7.09e-09	-0.028	5.22e-01
Monocyte	C3AR1	0.335	1.93e-15	-0.024	5.94e-01	0.043	3.29e-01	0.068	1.25e-01	0.482	3.01e-31	-0.186	2.25e-05
	CD86	0.196	5.04e-06	0.006	8.84e-01	0.05	2.61e-01	0.081	6.55e-02	0.496	0e+00	-0.166	1.51e-04
	CSF1R	0.243	1.28e-08	0.047	2.86e-01	0.041	3.53e-01	0.112	1.11e-02	0.497	1.94e-33	-0.112	1.1e-02
Neutrophils	CCR7	0.122	4.78e-03	0.199	5.42e-06	0.013	7.75e-01	0.18	4.29e-05	0.245	2.04e-08	0.072	1.04e-01
	ITGAM	0.238	2.56e-08	0.15	6.27e-04	0.009	8.4e-01	0.128	3.52e-03	0.496	0e+00	-0.058	1.91e-01
	SIGLEC5	0.359	1.16e-17	0.017	7.01e-01	0.049	2.7e-01	0.081	6.48e-02	0.398	6.03e-21	-0.117	7.89e-03
Dendritic cell	CD1C	0.259	1.21e-09	0.152	5.39e-04	-0.123	5.34e-03	0.155	4.08e-04	0.251	7.69e-09	0.039	3.79e-01
	HLA-DPA1	0.237	3.04e-08	0.21	1.59e-06	0.003	9.55e-01	0.197	6.78e-06	0.358	3.67e-17	-0.052	2.36e-01
	HLA-DPB1	0.149	5.82e-04	0.23	1.25e-07	-0.012	7.91e-01	0.255	4.15e-09	0.335	5.36e-15	-0.005	9.06e-01
	HLA-DQB1	0.152	4.37e-04	0.199	5.54e-06	0.062	1.6e-01	0.22	4.41e-07	0.297	6.18e-12	0.025	5.65e-01
	HLA-DRA	0.238	2.65e-08	0.167	1.47e-04	0.015	7.42e-01	0.194	9.13e-06	0.365	3.39e-18	-0.063	1.51e-01
	ITGAX	0.022	6.06e-01	0.276	1.91e-10	0.1	2.27e-01	0.256	3.95e-09	0.416	6.39e-23	0.108	1.42e-02
	NRP1	0.684	7.78e-75	-0.1	2.38e-02	-0.086	5.03e-02	0.025	5.7e-01	0.207	2.24e-06	-0.109	1.3e-02

Red mark means p < 0.05.

**Figure 7:**
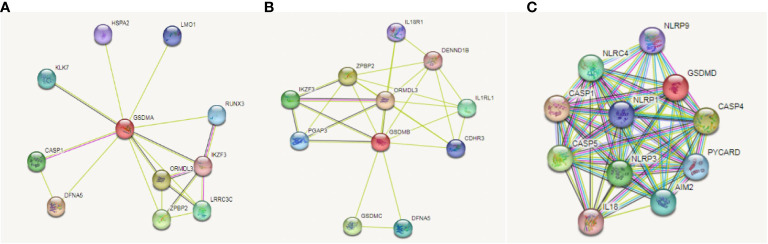
Correlation between GSDMs and immune check-points related genes in LUAD. We used the STRING database to analyze the correlation between GSDMA **(A)**, GSDMB **(B)**, and GSDMD **(C)** and immune related genes.

On the basis, we used STRING database to explore the relationship between gasdermin family and immune check-points related genes in LUAD to clarify whether this family could adjust tumorigenesis through immune related mechanism. We found that GSDMA (**Figure 7A**), GSDMB (**Figure 7B**), and GSDMD (**Figure 7C**) are correlated with immune related gene, including IKZF3, IL18R1, IL1RL1, IL18, NLRP9, and CASP5. Among them, NLRP9 and CASP5 could work as mediators of programmed cell death. So, we further speculated that GSDM family members play roles in immune related mechanism in LUAD.

## Discussion

The GSDM family regulates various physiological and pathological processes such as cell differentiation, coagulation, inflammation, and tumorigenesis, which is thought to be related to pyroptosis (36). Further studies have confirmed the irreplaceable role of GSDMs in tumor progression and development (37, 38). In recent years, LUAD gradually replaced lung squamous cell carcinoma as the most common subtype of malignant lung tumors (39).It is also the most common type of malignant tumor in the worldwide population (40).Currently, there are limited studies on GSDMs in LUAD. A study found that GSDMD deficiency reduces the cytolytic capacity of CD8+ T cells, and GSDMD is required for an optimal cytotoxic T lymphocytes response in cancer cells (41). The high expression of GSDMD may promote the occurrence and development of LUAD. This indicates a poor prognosis of LUAD and can be seen as an independent prognostic biomarker (21). Upregulated GSDMC expression is an independent indicator of poor FP and OS in patients with LUAD and is regarded as a promising predictor of prognosis (22). These studies confirmed the conclusions drawn from a series of databases used in the current study.

After comparing the expression levels of six GSDM in cancerous and normal tissues, this study revealed that the mRNA expression of GSDMs was significantly increased in LUAD, indicating that GSDMs may be involved in the tumorigenesis of LUAD. Our results showed that among the six members, GSDMD has the highest expression level, while GSDMC has the lowest expression level. Additionally, in terms of protein expression, IHC results showed that the expression of GSDMB, GSDMC, GSDMD, and GSDME in LUAD tissues was higher than that in normal tissues, particularly GSDMD. To validate this result, we performed IHC of GSDMD on 10 specimens. The experimental results support the search results from the database that GSDMD is indeed highly expressed in LUAD tissues. Next, a study on the prognosis of patients with LUAD, based on the expression levels of GSDMs, was conducted. The results showed that expression of GSDMs was elevated in most histological subtypes of LUAD. This further suggests that the abnormal expression of GSDMs has an important impact on the clinicopathological characteristics of different types of LUAD. The OS curves showed that the GSDMB, GSDMC, GSDMD, and GSDME were significantly associated with the poor prognosis of patients with LUAD. Their expression levels were almost all significantly elevated at different stages of LUAD. It was further found that the methylation levels of GSDMC and GSDMD in these four genes were decreased in LUAD. The mutation rates of GSDMC and GSDMD in LUAD were as high as 16% and 19%, respectively. Through KEGG enrichment analysis, we found that 10 potential signaling pathways were associated with GSDM, among which folic acid biosynthesis, bile secretion and IL-17 signaling pathways ranked the top three, suggesting that these pathways may be closely related to the progression of LUAD. Combined with previous studies, it was speculated that GSDMC and GSDMD have the potential to become new biomarkers and therapeutic targets for LUAD, and these two genes may deserve better and deeper exploration.

Based on the studies discussed, the cBioPortal database was used to explore the correlated genes of the GSDM family, and, thereafter, the WebGestalt database was used for functional enrichment analysis. This study demonstrated that some GSDM-associated pathways and biological processes are related to cellular immunity, which is crucial for tumorigenesis. Subsequently, the TIMER database was used to study several important immune cells and the relationship between the signature markers of these immune cells and the expression of GSDMs in LUAD. The results revealed that GSDM expression is correlated with various immune cells and most of their marker genes in LUAD. Later, we explored the relationship between the gasdermin family and immune checkpoint-related genes in LUAD to elucidate whether this family can regulate tumorigenesis through immune-related mechanisms. We found that GSDMA, GSDMB, and GSDMD were associated with immune-related genes. Among these genes, NLRP9 and CASP5 can act as mediators of programmed cell death. Therefore, we further speculated that GSDM family members play a role in the immune-related mechanisms of LUAD. We plan to explore the critical role of immune cell infiltration in antitumor immunity in the future.

## Conclusions

In conclusion, the present study assessed the molecular profiles of the GSDM family using bioinformatic databases. The results suggested that the expression of GSDMs (especially GSDMD) may be involved in the development and progression of LUAD. These findings may help enhance our understanding of LUAD and provide further details on prognosis prediction and treatment strategies for patients with LUAD.

## Data availability statement

The datasets presented in this study can be found in online repositories. The names of the repository/repositories and accession number(s) can be found in the article/**Supplementary Material.**

## Author contributions

C-LO and J-PW contributed to the conception and design of the study. L-SP and S-LD performed resource analysis, and wrote the first draft of the manuscript. All authors listed have made a substantial, direct, and intellectual contribution to the work and approved it for publication.

## Funding

This work was supported by the National Natural Science Foundation of China (No. 81602167 and 81903032), the China Postdoctoral Science Foundation (2020M672520), the Hunan Provincial Natural Science Foundation of China (No. 2021JJ31100 and 2021JJ4101), and the Science and Technology Program Foundation of Changsha City (No. kq2004085).

## Conflict of interest

The authors declare that the research was conducted in the absence of any commercial or financial relationships that could be construed as a potential conflict of interest.

## Publisher’s note

All claims expressed in this article are solely those of the authors and do not necessarily represent those of their affiliated organizations, or those of the publisher, the editors and the reviewers. Any product that may be evaluated in this article, or claim that may be made by its manufacturer, is not guaranteed or endorsed by the publisher.

## Abbreviations

GSDM, Gasdermin; LUAD, lung adenocarcinoma; GEPIA, gene expression profiling interactive analysis; THPA, the Human Protein Atlas; TIMER, tumor immune estimation resource; FP, first progression; OS, overall survival; KEGG, Encyclopedia of Genes and Genomes; GO, Gene Ontology.
