# Supplementary material for: Prognostic value and immune infiltration of the gasdermin family in lung adenocarcinoma
Source: Front Oncol. 2022 Nov 25;12:1043862. doi: 10.3389/fonc.2022.1043862 (PMC9732578; doi:10.3389/fonc.2022.1043862)
Supplement: Supplementary file 1 [file Table_1.docx]

**Supplementary Table 1**

| Correlated Gene | Cytoband | Spearman's Correlation | p-Value | q-Value |
| --- | --- | --- | --- | --- |
| TMEM86A | 11p15.1 | 0.402061 | 5.77E-21 | 1.15E-16 |
| MS4A4A | 11q12.2 | 0.398345 | 1.41E-20 | 1.41E-16 |
| SLCO2B1 | 11q13.4 | 0.389393 | 1.17E-19 | 7.78E-16 |
| ATP6V1B2 | 8p21.3 | 0.382595 | 5.57E-19 | 2.78E-15 |
| CD4 | 12p13.31 | 0.380918 | 8.15E-19 | 3.25E-15 |
| NCKAP1L | 12q13.13-q13.2 | 0.378975 | 1.26E-18 | 4.03E-15 |
| MS4A6A | 11q12.2 | 0.378469 | 1.41E-18 | 4.03E-15 |
| LAIR1 | 19q13.42 | 0.377348 | 1.82E-18 | 4.53E-15 |
| IGSF6 | 16p12.2 | 0.37536 | 2.82E-18 | 6.27E-15 |
| FOLR2 | 11q13.4 | 0.372342 | 5.49E-18 | 1.10E-14 |
| CD84 | 1q23.3 | 0.370685 | 7.89E-18 | 1.35E-14 |
| GAL3ST4 | 7q22.1 | 0.370552 | 8.12E-18 | 1.35E-14 |
| HAVCR2 | 5q33.3 | 0.368309 | 1.32E-17 | 2.03E-14 |
| GPNMB | 7p15.3 | 0.367675 | 1.51E-17 | 2.07E-14 |
| PLEKHO2 | 15q22.31 | 0.367561 | 1.55E-17 | 2.07E-14 |
| MS4A7 | 11q12.2 | 0.366053 | 2.15E-17 | 2.68E-14 |
| LAPTM5 | 1p35.2 | 0.364068 | 3.28E-17 | 3.64E-14 |
| C3AR1 | 12p13.31 | 0.362223 | 4.85E-17 | 5.10E-14 |
| DAB2 | 5p13.1 | 0.361653 | 5.47E-17 | 5.47E-14 |
| ADORA3 | 1p13.2 | 0.361256 | 5.95E-17 | 5.66E-14 |
| SIGLEC7 | 19q13.41 | 0.359545 | 8.53E-17 | 7.74E-14 |
| LCP1 | 13q14.13 | 0.358985 | 9.59E-17 | 8.33E-14 |
| SLC7A7 | 14q11.2 | 0.3575 | 1.31E-16 | 1.09E-13 |
| GPR34 | Xp11.4 | 0.356714 | 1.54E-16 | 1.20E-13 |
| FPR3 | 19q13.41 | 0.356514 | 1.60E-16 | 1.20E-13 |
| PIK3AP1 | 10q24.1 | 0.356444 | 1.63E-16 | 1.20E-13 |
| PTPRO | 12p12.3 | 0.356063 | 1.76E-16 | 1.26E-13 |
| CD28 | 2q33.2 | 0.355593 | 1.94E-16 | 1.34E-13 |
| CSF1R | 5q32 | 0.355305 | 2.06E-16 | 1.37E-13 |
| GPR137B | 1q42.3 | 0.353301 | 3.11E-16 | 2.00E-13 |
| C1QC | 1p36.12 | 0.351268 | 4.71E-16 | 2.87E-13 |
| CD53 | 1p13.3 | 0.35124 | 4.74E-16 | 2.87E-13 |
| CR1 | 1q32.2 | 0.350893 | 5.09E-16 | 2.99E-13 |
| CD300LF | 17q25.1 | 0.349726 | 6.45E-16 | 3.68E-13 |
| PLEK | 2p14 | 0.349544 | 6.69E-16 | 3.71E-13 |
| SELPLG | 12q24.11 | 0.34886 | 7.68E-16 | 3.95E-13 |
| KCNAB2 | 1p36.31 | 0.348843 | 7.70E-16 | 3.95E-13 |
| MPEG1 | 11q12.1 | 0.348834 | 7.72E-16 | 3.95E-13 |
| RNASE6 | 14q11.2 | 0.348618 | 8.06E-16 | 4.03E-13 |
| NFAM1 | 22q13.2 | 0.348239 | 8.70E-16 | 4.21E-13 |
| LRRC25 | 19p13.11 | 0.348149 | 8.86E-16 | 4.21E-13 |
| CD33 | 19q13.41 | 0.346358 | 1.27E-15 | 5.90E-13 |
| EVI2A | 17q11.2 | 0.346024 | 1.36E-15 | 6.16E-13 |
| TLR4 | 9q33.1 | 0.345889 | 1.39E-15 | 6.19E-13 |
| TRPV2 | 17p11.2 | 0.34567 | 1.46E-15 | 6.32E-13 |
| SASH3 | Xq26.1 | 0.34517 | 1.61E-15 | 6.72E-13 |
| SLAMF8 | 1q23.2 | 0.345149 | 1.62E-15 | 6.72E-13 |
| SCIMP | 17p13.2 | 0.344628 | 1.79E-15 | 7.30E-13 |
| CD86 | 3q13.33 | 0.344318 | 1.91E-15 | 7.61E-13 |
| BTK | Xq22.1 | 0.343775 | 2.12E-15 | 8.31E-13 |
| C1QB | 1p36.12 | 0.342496 | 2.73E-15 | 1.05E-12 |
| FERMT3 | 11q13.1 | 0.341985 | 3.02E-15 | 1.14E-12 |
| SIGLEC9 | 19q13.3-q13.4 | 0.341672 | 3.21E-15 | 1.19E-12 |
| SLC37A2 | 11q24.2 | 0.341286 | 3.47E-15 | 1.26E-12 |
| SPI1 | 11p11.2 | 0.340958 | 3.70E-15 | 1.31E-12 |
| PIK3R5 | 17p13.1 | 0.340885 | 3.75E-15 | 1.31E-12 |
| LAT2 | 7q11.23 | 0.340826 | 3.80E-15 | 1.31E-12 |
| CD163 | 12p13.31 | 0.339913 | 4.54E-15 | 1.53E-12 |
| CYTH4 | 22q13.1 | 0.339856 | 4.59E-15 | 1.53E-12 |
| MNDA | 1q23.1 | 0.339771 | 4.67E-15 | 1.53E-12 |
| TFEC | 7q31.2 | 0.339451 | 4.97E-15 | 1.60E-12 |
| CTSB | 8p23.1 | 0.338594 | 5.87E-15 | 1.86E-12 |
| DOK2 | 8p21.3 | 0.337267 | 7.59E-15 | 2.36E-12 |
| IL10 | 1q32.1 | 0.337213 | 7.67E-15 | 2.36E-12 |
| P2RX7 | 12q24.31 | 0.336672 | 8.52E-15 | 2.58E-12 |
| LY86 | 6p25.1 | 0.336457 | 8.88E-15 | 2.65E-12 |
| MPP1 | Xq28 | 0.335867 | 9.95E-15 | 2.92E-12 |
| VSIG4 | Xq12 | 0.335488 | 1.07E-14 | 3.07E-12 |
| STAC3 | 12q13.3 | 0.33545 | 1.08E-14 | 3.07E-12 |
| F13A1 | 6p25.1 | 0.335124 | 1.15E-14 | 3.23E-12 |
| ITGB2 | 21q22.3 | 0.334472 | 1.30E-14 | 3.61E-12 |
| ACP5 | 19p13.2 | 0.3344 | 1.32E-14 | 3.61E-12 |
| AOAH | 7p14.2 | 0.333986 | 1.43E-14 | 3.85E-12 |
| SLC2A9 | 4p16.1 | 0.333597 | 1.54E-14 | 4.09E-12 |
| NCF2 | 1q25.3 | 0.33315 | 1.67E-14 | 4.40E-12 |
| AIF1 | 6p21.33 | 0.33307 | 1.70E-14 | 4.41E-12 |
| TMEM273 | 10q11.23 | 0.332925 | 1.75E-14 | 4.47E-12 |
| CCRL2 | 3p21.31 | 0.332866 | 1.77E-14 | 4.47E-12 |
| TYROBP | 19q13.12 | 0.332652 | 1.84E-14 | 4.60E-12 |
| DOCK2 | 5q35.1 | 0.332552 | 1.88E-14 | 4.63E-12 |
| SIGLEC14 | 19q13.41 | 0.332254 | 1.99E-14 | 4.84E-12 |
| TLR7 | Xp22.2 | 0.331715 | 2.20E-14 | 5.26E-12 |
| TLR8 | Xp22.2 | 0.331656 | 2.22E-14 | 5.26E-12 |
| EVI2B | 17q11.2 | 0.331625 | 2.24E-14 | 5.26E-12 |
| DNAJC5B | 8q13.1 | 0.33097 | 2.53E-14 | 5.88E-12 |
| AGAP2 | 12q14.1 | 0.330463 | 2.79E-14 | 6.35E-12 |
| PILRA | 7q22.1 | 0.330443 | 2.80E-14 | 6.35E-12 |
| IFI30 | 19p13.11 | 0.329774 | 3.17E-14 | 7.12E-12 |
| FGD2 | 6p21.2 | 0.329703 | 3.22E-14 | 7.14E-12 |
| TREM2 | 6p21.1 | 0.329087 | 3.61E-14 | 7.93E-12 |
| IRF8 | 16q24.1 | 0.32844 | 4.08E-14 | 8.85E-12 |
| C1QA | 1p36.12 | 0.327862 | 4.54E-14 | 9.76E-12 |
| CYBB | Xp21.1-p11.4 | 0.327508 | 4.85E-14 | 1.03E-11 |
| GPR65 | 14q31.3 | 0.326674 | 5.67E-14 | 1.19E-11 |
| MYO1F | 19p13.2 | 0.326298 | 6.08E-14 | 1.27E-11 |
| EMILIN2 | 18p11.32-p11.31 | 0.326091 | 6.32E-14 | 1.29E-11 |
| IL10RA | 11q23.3 | 0.326085 | 6.33E-14 | 1.29E-11 |
| SLC31A2 | 9q32 | 0.326022 | 6.40E-14 | 1.29E-11 |
| RGS18 | 1q31.2 | 0.325667 | 6.84E-14 | 1.37E-11 |
| HLA-DMB | 6p21.32 | 0.325595 | 6.93E-14 | 1.37E-11 |
| LILRB4 | 19q13.42 | 0.324987 | 7.76E-14 | 1.52E-11 |
| MSR1 | 8p22 | 0.32494 | 7.82E-14 | 1.52E-11 |
| CRYBB1 | 22q12.1 | 0.324833 | 7.98E-14 | 1.52E-11 |
| NRROS | 3q29 | 0.324818 | 8.00E-14 | 1.52E-11 |
| PLXDC2 | 10p12.31 | 0.324354 | 8.72E-14 | 1.64E-11 |
| CMKLR1 | 12q23.3 | 0.324046 | 9.23E-14 | 1.72E-11 |
| MRC1 | 10p12.33 | 0.323846 | 9.57E-14 | 1.77E-11 |
| LILRA6 | 19q13.42 | 0.323742 | 9.76E-14 | 1.79E-11 |
| TNFAIP8L2 | 1q21.3 | 0.323672 | 9.89E-14 | 1.80E-11 |
| FCER1G | 1q23.3 | 0.323039 | 1.11E-13 | 1.99E-11 |
| ACSM5 | 16p12.3 | 0.323019 | 1.11E-13 | 1.99E-11 |
| P2RY13 | 3q25.1 | 0.322736 | 1.17E-13 | 2.08E-11 |
| LCP2 | 5q35.1 | 0.322458 | 1.24E-13 | 2.16E-11 |
| MAFB | 20q12 | 0.32237 | 1.26E-13 | 2.18E-11 |
| TM6SF1 | 15q25.2 | 0.322184 | 1.30E-13 | 2.24E-11 |
| DPEP2 | 16q22.1 | 0.321695 | 1.42E-13 | 2.43E-11 |
| SIRPB2 | 20p13 | 0.32129 | 1.53E-13 | 2.59E-11 |
| SIGLEC1 | 20p13 | 0.320986 | 1.62E-13 | 2.71E-11 |
| ALOX5 | 10q11.21 | 0.3209 | 1.64E-13 | 2.73E-11 |
| CPVL | 7p14.3 | 0.320324 | 1.82E-13 | 2.99E-11 |
| ITGAM | 16p11.2 | 0.320089 | 1.90E-13 | 3.07E-11 |
| ARHGAP30 | 1q23.3 | 0.320078 | 1.91E-13 | 3.07E-11 |
| CD209 | 19p13.2 | 0.319896 | 1.97E-13 | 3.15E-11 |
| LIPA | 10q23.31 | 0.319668 | 2.05E-13 | 3.26E-11 |
| FGR | 1p35.3 | 0.31951 | 2.11E-13 | 3.33E-11 |
| RUBCNL | 13q14.13 | 0.31946 | 2.13E-13 | 3.33E-11 |
| MAF | 16q23.2 | 0.319302 | 2.20E-13 | 3.40E-11 |
| OTOA | 16p12.2 | 0.318671 | 2.46E-13 | 3.76E-11 |
| WAS | Xp11.23 | 0.318667 | 2.46E-13 | 3.76E-11 |
| TMEM150B | 19q13.42 | 0.318461 | 2.56E-13 | 3.84E-11 |
| LILRB3 | 19q13.42 | 0.318452 | 2.56E-13 | 3.84E-11 |
| LPXN | 11q12.1 | 0.318426 | 2.57E-13 | 3.84E-11 |
| PTAFR | 1p35.3 | 0.318149 | 2.70E-13 | 4.00E-11 |
| AP1S2 | Xp22.2 | 0.318111 | 2.72E-13 | 4.00E-11 |
| SNX20 | 16q12.1 | 0.317754 | 2.90E-13 | 4.23E-11 |
| P2RY12 | 3q25.1 | 0.317718 | 2.92E-13 | 4.23E-11 |
| CSF2RB | 22q12.3 | 0.317587 | 2.99E-13 | 4.30E-11 |
| HCK | 20q11.21 | 0.317277 | 3.16E-13 | 4.51E-11 |
| TNFSF8 | 9q32-q33.1 | 0.31694 | 3.36E-13 | 4.76E-11 |
| SLC1A3 | 5p13.2 | 0.316612 | 3.57E-13 | 5.02E-11 |
| CD68 | 17p13.1 | 0.315985 | 3.99E-13 | 5.57E-11 |
| CD300C | 17q25.1 | 0.315851 | 4.09E-13 | 5.67E-11 |
| PARVG | 22q13.31 | 0.314842 | 4.89E-13 | 6.74E-11 |
| NPL | 1q25.3 | 0.31474 | 4.98E-13 | 6.82E-11 |
| NLRC4 | 2p22.3 | 0.314607 | 5.10E-13 | 6.93E-11 |
| CORO1A | 16p11.2 | 0.314391 | 5.30E-13 | 7.16E-11 |
| CD37 | 19q13.33 | 0.313015 | 6.77E-13 | 9.07E-11 |
| SIRPA | 20p13 | 0.312944 | 6.85E-13 | 9.13E-11 |
| CD163L1 | 12p13.31 | 0.312607 | 7.27E-13 | 9.62E-11 |
| HLA-DOA | 6p21.32 | 0.312156 | 7.88E-13 | 1.03E-10 |
| ITGAX | 16p11.2 | 0.312104 | 7.95E-13 | 1.03E-10 |
| FCGR3A | 1q23.3 | 0.312094 | 7.96E-13 | 1.03E-10 |
| CD14 | 5q31.3 | 0.312089 | 7.97E-13 | 1.03E-10 |
| LGALS9 | 17q11.2 | 0.311868 | 8.29E-13 | 1.06E-10 |
| GIMAP4 | 7q36.1 | 0.310646 | 1.03E-12 | 1.30E-10 |
| SLC15A3 | 11q12.2 | 0.310625 | 1.03E-12 | 1.30E-10 |
| IL2RA | 10p15.1 | 0.310102 | 1.13E-12 | 1.42E-10 |
| GNAI2 | 3p21.31 | 0.309865 | 1.18E-12 | 1.47E-10 |
| HLA-DPA1 | 6p21.32 | 0.309735 | 1.20E-12 | 1.49E-10 |
| APOC2 | 19q13.32 | 0.309385 | 1.28E-12 | 1.58E-10 |
| SLA | 8q24.22 | 0.309019 | 1.37E-12 | 1.67E-10 |
| PTPRC | 1q31.3-q32.1 | 0.308873 | 1.40E-12 | 1.71E-10 |
| MFSD1 | 3q25.32 | 0.308307 | 1.55E-12 | 1.85E-10 |
| CARD9 | 9q34.3 | 0.308291 | 1.55E-12 | 1.85E-10 |
| IL12RB1 | 19p13.11 | 0.308272 | 1.55E-12 | 1.85E-10 |
| MFNG | 22q13.1 | 0.308264 | 1.56E-12 | 1.85E-10 |
| GM2A | 5q33.1 | 0.308178 | 1.58E-12 | 1.87E-10 |
| LILRB5 | 19q13.4 | 0.308138 | 1.59E-12 | 1.87E-10 |
| CLEC4A | 12p13.31 | 0.307489 | 1.78E-12 | 2.08E-10 |
| CCR5 | 3p21.31 | 0.306203 | 2.22E-12 | 2.58E-10 |
| ATP8B4 | 15q21.2 | 0.306141 | 2.25E-12 | 2.59E-10 |
| ZEB2 | 2q22.3 | 0.306112 | 2.26E-12 | 2.59E-10 |
| LSP1 | 11p15.5 | 0.306027 | 2.29E-12 | 2.62E-10 |
| ABI3 | 17q21.32 | 0.30578 | 2.39E-12 | 2.72E-10 |
| CD5 | 11q12.2 | 0.305706 | 2.42E-12 | 2.73E-10 |
| PDCD1LG2 | 9p24.1 | 0.305452 | 2.53E-12 | 2.84E-10 |
| WIPF1 | 2q31.1 | 0.305368 | 2.57E-12 | 2.87E-10 |
| STK10 | 5q35.1 | 0.305334 | 2.58E-12 | 2.87E-10 |
| PLA2G15 | 16q22.1 | 0.304924 | 2.77E-12 | 3.06E-10 |
| GIMAP6 | 7q36.1 | 0.3047 | 2.88E-12 | 3.16E-10 |
| SIGLEC8 | 19q13.33-q13.41 | 0.304118 | 3.18E-12 | 3.46E-10 |
| SH2B3 | 12q24.12 | 0.304107 | 3.19E-12 | 3.46E-10 |
| TMOD2 | 15q21.2 | 0.303997 | 3.25E-12 | 3.51E-10 |
| FCGR2A | 1q23.3 | 0.303843 | 3.34E-12 | 3.58E-10 |
| HLA-DPB1 | 6p21.32 | 0.303805 | 3.36E-12 | 3.59E-10 |
| NLRP3 | 1q44 | 0.30365 | 3.45E-12 | 3.66E-10 |
| CEACAM21 | 19q13.2 | 0.303607 | 3.47E-12 | 3.67E-10 |
| ARHGEF6 | Xq26.3 | 0.303551 | 3.51E-12 | 3.69E-10 |
| FCGR1A | 1q21.2 | 0.303264 | 3.68E-12 | 3.85E-10 |
| LILRB1 | 19q13.42 | 0.302988 | 3.86E-12 | 4.01E-10 |
| KCNK13 | 14q32.11 | 0.302936 | 3.89E-12 | 4.02E-10 |
| PDE6G | 17q25.3 | 0.30292 | 3.90E-12 | 4.02E-10 |
| NCF4 | 22q12.3 | 0.302843 | 3.96E-12 | 4.05E-10 |
| RTN1 | 14q23.1 | 0.301901 | 4.64E-12 | 4.73E-10 |
| FOXP3 | Xp11.23 | 0.301855 | 4.68E-12 | 4.74E-10 |
| SPN | 16p11.2 | 0.301632 | 4.86E-12 | 4.90E-10 |
| ADAP2 | 17q11.2 | 0.301512 | 4.96E-12 | 4.98E-10 |
| LILRA4 | 19q13.42 | 0.301476 | 4.99E-12 | 4.98E-10 |
| CD80 | 3q13.33 | 0.30095 | 5.45E-12 | 5.42E-10 |
| GLIPR1 | 12q21.2 | 0.300925 | 5.48E-12 | 5.42E-10 |
| BIN2 | 12q13.13 | 0.30063 | 5.76E-12 | 5.64E-10 |
| IKZF1 | 7p12.2 | 0.300629 | 5.76E-12 | 5.64E-10 |
| PIK3R6 | 17p13.1 | 0.300553 | 5.83E-12 | 5.68E-10 |
| FAM78A | 9q34.13 | 0.300494 | 5.89E-12 | 5.71E-10 |
| CD180 | 5q12.3 | 0.300311 | 6.07E-12 | 5.86E-10 |
